# Knee proprioception in children with spastic cerebral palsy: an observational study

**DOI:** 10.1186/s12887-026-07652-2

**Published:** 2026-09-15

**Authors:** Annika Ericson, Åsa Bartonek, Israel Luis, Marie Eriksson, Cecilia Lidbeck

**Affiliations:** 1 Division of Paediatric Neurology, Department of Women’s and Children’s Health, Karolinska Institutet, Stockholm, Sweden; 2 Astrid Lindgren Children’s Hospital, Karolinska University Hospital, Stockholm, Sweden; 3 Department of Engineering Mechanics, KTH MoveAbility, , KTH Royal Institute of Technology, Stockholm, Sweden; 4 Division of Paediatric Neurology, Motion Analysis Laboratory, QA:27, Karolinska Institutet, Karolinska vägen 37 A, Stockholm, 171 76 Sweden

**Keywords:** Cerebral palsy, Proprioception, Sensorimotor, Sensory processing

## Abstract

**Background:**

Proprioception, the sense of body position and movement, is vital for motor control but has been sparsely explored in children with cerebral palsy (CP), particularly in the lower extremities. This study explored proprioception as the capacity to reproduce and maintain a knee flexion angle in children with CP.

**Methods:**

Fifty-seven children aged 6–18 years with spastic CP; 20 unilateral (USCP), and 37 bilateral (BSCP), at Gross Motor Function Classification System (GMFCS) I-IV, and 42 typically developing (TD) children participated. Three-dimensional motion analysis was used to capture knee movements during a joint position sense test performed in sitting with occluded vision. The test consisted of (i) the criterion angle (CA) trial; the leg was passively elevated to a knee flexion angle of 45 degrees (CA) by an examiner and the position was maintained by the child, and (ii) the reproduced angle (RA) trial; the angle was actively reproduced (RA) and maintained by the child. Joint position sense errors were evaluated as the difference between CA and RA. The children’s capacity to maintain the leg position was measured as the difference in knee angles, fluctuations (root mean square error) and drift (linear regression). Data were analysed with non-parametric statistics.

**Results:**

Joint position sense errors were greater in the USCP-group than the BSCP-group (*p* = 0.037), with a more extended knee in the USCP-group (*p* = 0.012). When maintaining the passively positioned knee angle for 2.5 s, the CP-group compared to the TD-group, showed increased knee flexion and greater movement fluctuations (*p* = 0.006). Within-group analysis showed increased knee flexion at the passively assumed position (CA vs. maintained for 2.5 s) compared to at the actively assumed position (RA vs. maintained for 2.5 s) in the BSCP-group (*p* = 0.015) and the GMFCS II-group (*p* = 0.028). The BSCP-group also showed increased drift over time towards knee flexion when the leg was passively compared to actively positioned (*p* = 0.040).

**Conclusions:**

The results reveal a divergence in proprioceptive function: while the USCP-group had greater difficulty than the BSCP-group in reproducing a knee angle, the BSCP-group demonstrated greater difficulty in maintaining the knee position during passive positioning of the lower limb.

**Trial registration:**

This study was observational and was therefore not registered as a clinical trial.

**Supplementary Information:**

The online version contains supplementary material available at https://doi.org/10.1186/s12887-026-07652-2.

## Background

In daily activities, we rely on signals from our body in motion to respond to spatial demands and changes in the environment [1]. In individuals with cerebral palsy (CP), a condition characterized by disorders of movement and posture, disturbances in detecting and interpreting sensory information may affect motor functioning [2].

Cerebral palsy is an early-onset neurodevelopmental condition attributed to dysplasia or injury of the foetal or infant brain, resulting in activity limitations [2, 3]. In a newly proposed description of CP, it was emphasized that the sensorimotor control of movement and posture is a core feature [3]. The constraints imposed by activity limitations may further hinder exploration of the environment, thereby influencing learning and perceptual experiences and subsequently exacerbating the sensorimotor disorders [2, 3]. Based on the predominant neurological symptoms and the motor involvement, the spastic type of CP can be classified as unilateral, USCP (mainly involving limbs on one side of the body), or bilateral, BSCP (involving limbs on both sides of the body) [4]. Motor functioning is commonly classified with the Gross Motor Functioning Classification System (GMFCS-E&R) on a I-V scale, where individuals at GMFCS level I walk independently in all environments, and those functioning at level V need assistance with all transfers [5]. Individuals with CP exhibit a range of motor limitations with variability in standing [6] and walking e.g., excessively flexed or hyperextended knees during gait [7]. The functional limitations may be attributed to underlying impairments such as muscle weakness [8], poor motor control [3], impaired sensory processing [2, 3], or a combination of these. Distinguishing between the impairments’ influence on motor activity performance is complex but important for understanding the posture and movement disorders of CP and for planning of individualized interventions.

Sensory disturbances, such as a perceptual disorder affecting posture and body position, have been described by parents of children with BSCP [7], particularly in those with more limited gross motor functioning (GMFCS III-IV) [9]. Previous research has shown that children with CP tend to rely more on vision for postural orientation in sitting [10], standing [11], and walking [12] than typically developing (TD) children, possibly due to deficits in the somatosensory system. Proprioceptive disturbances have been observed in both children with BSCP [13] and USCP [13, 14] as compared to TD children. However, in children with USCP, the upper extremities are more commonly examined [15].

Sherrington introduced the term ”proprioception” [16], which is currently described as the sensory modalities responsible for detecting static joint positions (statesthesia), joint movements (kinaesthesia), and dynamic changes in joint orientation [17, 18]. Proprioception encompasses the perception of body positions and movement, the sense of tension or force, and the sense of effort [19]. Sensory stimuli are detected by specialized receptors in the muscles, tendons, joints, and skin [20, 21]. Neural pathways transmit the information to the central nervous system for processing, which helps to adjust and anticipate movements [18], such as adjusting step length when walking on uneven ground or ascending and descending stairs without looking at the legs.

In children with spastic CP, deficits in hip joint-position sense and kinaesthesia were found in comparison to a TD group [13] and in a later study, deficits in hip proprioception were associated with increased postural sway and reduced gait speed [22]. Moreover, in a small group of ambulatory children with BSCP, greater errors in reproducing target ankle joint positions were associated with lower scores on the subdomain ‘postural responses’ in the Balance Evaluation Systems test [23]. In a recent study, the proprioceptive function of the ankles, knees, and hips in a group of children with upper motor neuron lesions (including children with CP) differed from TD peers when examined in sitting [24].

In contrast to the above-mentioned findings, which indicate deficits in lower-limb proprioception in children with CP, McLaughlin and colleagues found no differences between children with CP and TD peers in the capacity to actively reproduce knee angles in sitting without visual input of the legs [25]. Similarly, recent findings from our research group demonstrated that children with BSCP could actively reproduce knee flexion angles with accuracy comparable to TD peers as measured with a joint position sense (JPS) test [26] and three-dimensional motion analysis (3DMA) in sitting with occluded vision [27]. However, greater difficulty in maintaining a passively induced knee position compared to an actively induced knee position was observed in the group with BSCP [27]. These findings underscore the need for further investigation into lower-limb proprioception in children with both spastic subtypes; BSCP and USCP.

The general aim of this study was to further explore knee proprioception in children with BSCP and USCP compared to a TD reference group. More specifically, to investigate the ability to reproduce and maintain a knee angle during a JPS test. The hypothesis was that there would be no differences in the capacity to reproduce the target angle between the groups. A further hypothesis was that maintaining a passively assumed knee angle would be more difficult for children with BSCP, and for those with more limited gross motor functioning, than for those with USCP, those walking independently in all environments (GMFCS I), and the TD group.

## Methods

This cross-sectional observational study exploring knee proprioception in children with CP referred to the neuro-paediatric department was conducted at Karolinska University Hospital, Stockholm, Sweden, between January 2019 and August 2020.

### Participants

Participants were 57 children, 20 girls and 37 boys, aged 6–18 years, with spastic CP. The CP group was further divided into subgroups: spastic subtypes; USCP: 20, BSCP: 37; and GMFCS groups: I: 31, II: 11, and III-IV: 15 (Table 1). The inclusion criteria for participation were sitting independently (arm support allowed), range of motion of the knee to allow for execution of the test, grade 4 or 5 on manual muscle testing of the knee extensors (0–5 scale) [28], compliance to perform the test and ability to understand the verbal instructions. The exclusion criterion was orthopaedic surgery in the knee within a year before participation. For comparison, a convenience sample of 42 TD children, 22 girls and 20 boys, aged 6–18 years participated (Table 1).

**Table 1:** Characteristics of the children typically developing (TD) and with cerebral palsy (CP). The children with CP are described with respect to spastic subtype: unilateral (USCP) and bilateral (BSCP) and with respect to Gross Motor Functioning Classification System (GMFCS) groups; I, II and III-IV

	TD (*n =* 42)	CP (*n =* 57)					
			USCP (*n =* 20)	BSCP (*n =* 37)	GMFCS I (*n* = 31)	GMFCS II (*n* = 11)	GMFCS III-IV (*n* = 15)
Age; median [min, max] years	10.9 [6.7, 18.0]	12.7 [6.3, 18.0]	9.9, [6.3, 17.7]	14.4 [6.4, 18.0]	10.3 [6.3, 18.0]	12.9 [9.4, 17.8]	15.6 [9.5, 17.7]
Height; median [min, max] cm	152.5 [118.0, 89.8]	152.0 [113.1, 180.0]	141.2 [117.0, 180.0]	154.0 [113.1, 178.0]	146.0 [113.1, 180.0]	151.0 [133.0, 173.0]	161.0 [130.0, 175.0]
Weight; median [min, max] kg	39.5 [22.1, 84.5]	45.6^1^ [21.0, 73.0]	39.2 [22.3, 60.1]	49.2^1^ [21.0, 73.0]	39.3 [21.0, 60.1]	43.6 [30.4, 57.0]	53.3^1^ [22.6, 73.0
GMFCS levels, number of children	N/A	I: 31, II:11, III: 13, IV:2	I:19, II:1	I:12, II:10, III:13, IV: 2	N/A	N/A	N/A

^1^Indicates missing data from one child, and *N/A *not applicable

### Reproducing the knee angle/joint position sense test

**Fig. 1:**
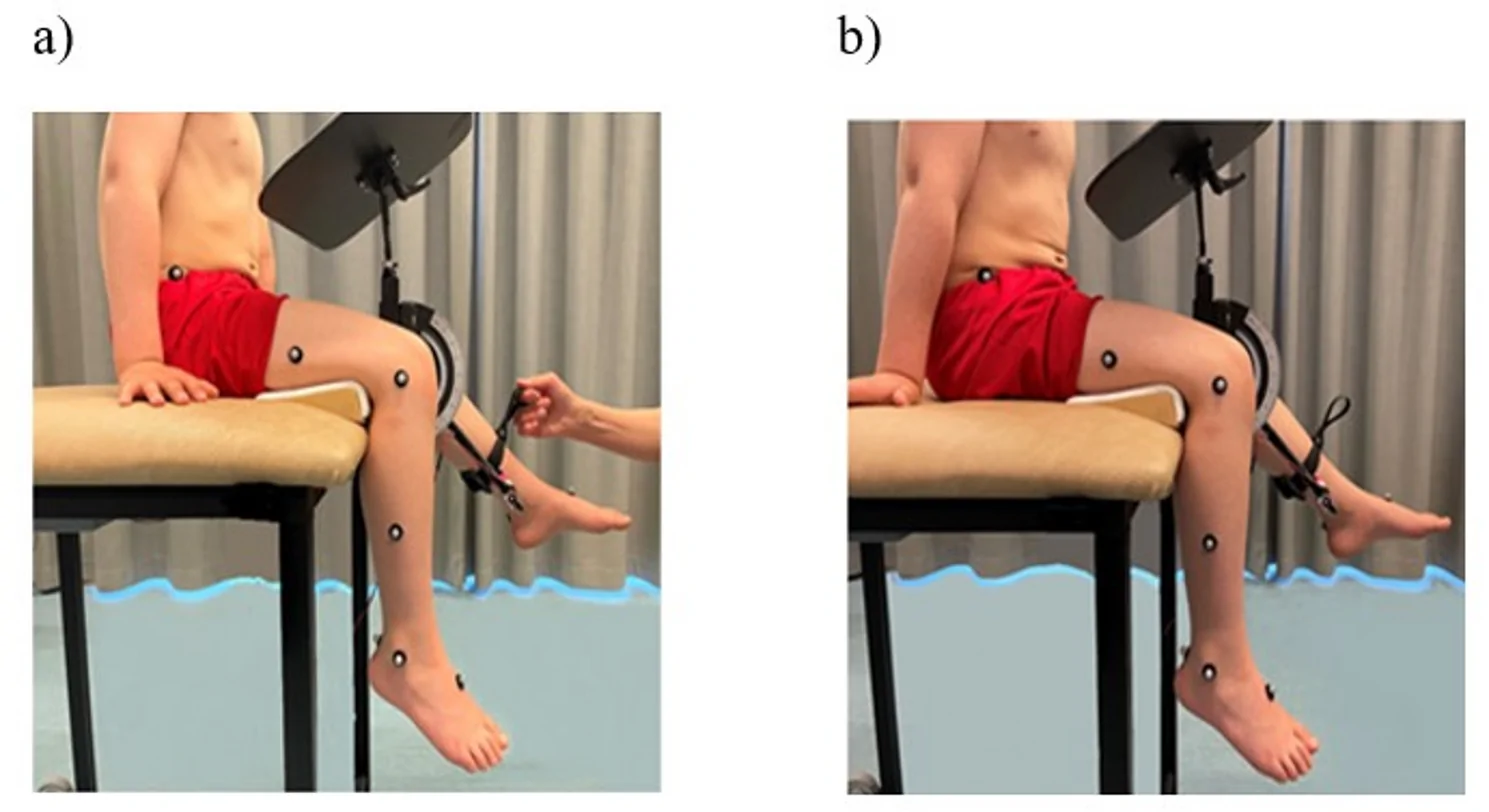
Picture modified from Bartonek et al. [27], showing the two trials in the knee movements task: **a**) criterion angle (CA) trial and **b**) reproduced angle (RA) trial

All participants were evaluated with a joint position sense (JPS) test during a knee movement task performed in sitting. The test measures the capacity to actively reproduce a leg position [26]. During testing the child sat on a customized stool with a 60 × 60 cm area and a height of 70 cm. To prevent visual information of the task, a height-adjustable tray table covered the children’s legs (Fig. 1).

The task consisted of two trials (i) the criterion angle-trial (CA-trial) in which the leg was passively elevated in a strap by an examiner to a CA of 45 degrees knee flexion, and (ii) the reproduced angle-trial (RA-trial) in which the child actively reproduced the angle (RA). To set the CA the examiner was guided by a red laser pointer attached to a goniometer placed on the stool (Fig. 1a). After the examiner had set the CA and the child maintained the leg itself, the strap was released (SR). The participant was asked to maintain the position for three seconds and memorize the position before returning to a resting position. During the RA-trial the participant was asked to actively reproduce the CA by extending the leg, yielding the RA, and maintain the position for three seconds (Fig. 1b).

The left and right legs were tested in a randomized order. The knee movement task was performed twice on each leg, with approximately 10 s of rest between. Verbal instructions were given, and a familiarization trial was undertaken to ensure understanding of the task. Each test session was conducted by two out of four examiners. All examiners received training to ensure consistent administration.

### Maintaining the knee angle

**Fig. 2:**
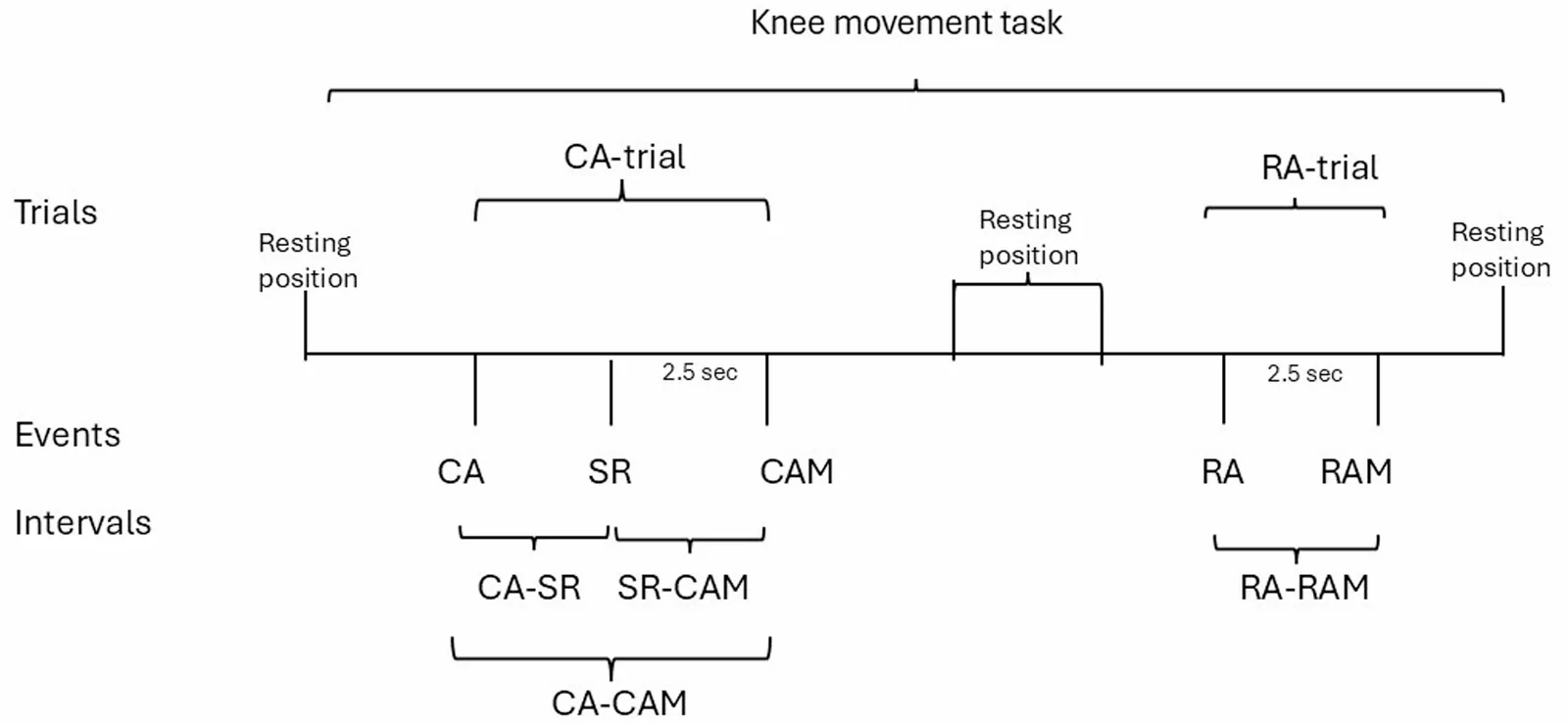
Illustration of the knee movement task with the two trials (modified from Bartonek et al [27]); criterion angle (CA) trial and reproduced angle (RA) trial. The events: criterion angle (CA), strap release (SR), criterion angle maintained 2.5 seconds (CAM), reproduced angle (RA) and reproduced angle maintained 2.5 seconds (RAM). The intervals in the CA-trial: CA-SR, SR-CAM, CA-CAM and the interval in the RA trial: RA-RAM

In the passively induced CA-trial, the knee position was evaluated in relation to CA and the strap release (SR), i.e. CA/SR, SR/CA maintained 2.5 s (CAM), and CA/CAM. In the actively induced RA-trial, the knee position was evaluated at RA/RA maintained 2.5 s (RAM) (Fig. 2).

### Three-dimensional motion analysis

Knee movements and positions were captured with 3DMA, using 8 infra-red optical cameras with a sampling rate of 100 Hz (Vicon MX40^®^ Oxford Metrics, UK) and synchronized video recordings. A full-body kinematic, Plug-in-gait, Newington model [29] with 34 reflective markers attached to anatomical landmarks on the head, trunk, pelvis, and lower limbs was utilized, and markers were placed on the child in a sitting position.

### Data analysis

Data from the right leg were analysed for the TD children and for the group with BSCP, or from the left leg in case of missing data, and from the affected leg for the USCP group. Data were analysed from the second of the two knee movement tasks, or from the first task when data were missing.

In Vicon Nexus software (2.1) the event of strap release (SR) was manually marked with guidance of video observations. A custom-made MATLAB script (Version 2021a, The MathWorks, Inc., Natick, Massachusetts, US) with the Statistics and Machine Learning Toolbox was used to automatically detect and set the knee angles at CA and RA at movements of ≤ 2 degrees/second. In addition, CAM and RAM were automatically set 2.5 s after SR and RA, respectively (Fig. 2).

Joint position sense errors (absolute and relative errors) were calculated as the difference between the knee angle at CA and RA [30]. A positive (+) relative error indicates that the knee angle was overestimated/more extended in relation to the target angle. A negative (-) relative error indicates that the knee angle was underestimated/more flexed in relation to the target angle [30].

To evaluate the capacity to maintain the position of the lower limb in the CA trial and the RA trial, the difference in knee angles and the direction of the difference (considering the arithmetic sign) were calculated between events (Fig. 2). In the CA-trial, the duration (in seconds) from CA to SR was also measured. Moreover, deviations in the knee angle between intervals in the respective trials were calculated, hereafter referred to as fluctuations and drift of the position. Fluctuations of the knee position over time were measured with a calculation of root mean square error (RMSE) [degrees], reflecting the average magnitude of the error between predicted and actual values, calculated as:

$$\mathrm{RMSE}=\sqrt[2]{\textstyle\frac1{n}\sum\limits_{t=1}^{n}\left({\theta}_{{k}_{(t)}}-{\theta}_{{k}_{(t=0}}\right)^2}$$

Where ${\mathrm\theta}_{{\mathrm k}_{\left(\mathrm t\right)}}$is the knee angle at time *t*, and * n* is the number of time intervals. An RMSE of zero degrees reflects no fluctuation of the position, while a greater number reflects a greater fluctuation/error of the knee position (relative to the targeted position).

The drift in knee position over time was quantified as the regression slope. The regression slope was calculated with the following equation:

$${\widehat{\theta}}_{{k}_{\left(t\right)}}=a+b*t+e$$

Where ${\widehat{\theta\:}}_{{k}_{\left(t\right)}}$is the estimated knee joint angle [degrees], *b* is the regression slope [degrees/seconds], *a* the intercept [degrees], *t* the time [seconds], and *e* the error term [degrees]. The terms of the linear regression are found to minimize the least-squares difference between the estimated ${\widehat{\theta\:}}_{{k}_{\left(t\right)}}$and the experimental knee angle (${{\theta\:}_{k}}_{\left(t\right)}$). The linear regression was chosen based on visual inspection of the collected data, as there were no rapid (non-linear) changes in the knee angle. A value of zero indicates that there is no drift in the knee position. A positive value indicates that the knee flexion angle is increasing (drifting towards flexion) over time (degrees/second), and a negative value indicates that the knee flexion angle is decreasing (drifting towards extension) over time.

### Statistical analysis

Descriptive data are presented as numbers, median, minimum and maximum [min, max] values. Nonparametric statistics were used because the data were not normally distributed, as assessed by the Shapiro-Wilk test. To analyse possible between-group differences in age, height, weight, knee angle during the knee movement task, and time to strap release, a Kruskal-Wallis test with Mann-Whitney U post hoc tests was used for the GMFCS groups (I, II, III-IV), and Mann-Whitney U tests for the group with CP vs. TD and USCP vs. BSCP. The Bonferroni test was used to correct for multiple comparisons. To analyse possible within-group differences between knee angles in the CA-trial vs. the RA-trial, a Wilcoxon signed-rank test was applied. To analyse possible relationships between age and JPS error in the entire sample and for the CP group, a Spearman correlation was used. If significant, the following interpretation was used for the size of the agreement r_s_: 0.00-0.30, little/if any correlation, 0.30–0.50, low correlation, 0.50–0.70, moderate correlation, 0.70–0.90, high correlation, and 0.90-1.00, very high correlation [31]. Statistical analyses were performed using IBM SPSS Statistics version 30 (Chicago, IL, USA). The significance level was set at < 0.05.

## Results

### Participants

Age, height, and weight did not differ between the group with CP (*n* = 57) and the TD group (*n* = 42). With respect to spastic subtype, the children with BSCP compared to USCP were older (*p* = 0.001), taller (*p* = 0.019), and heavier (*p* = 0.019). The children at GMFCS III-IV compared to those at GMFCS I were older (*p* = 0.012), taller (*p* = 0.016), and heavier (*p* = 0.006), and compared to those at GMFCS II, they were heavier (*p* = 0.021) (Table 1).

### Reproducing the knee angle/joint position sense

**Fig. 3:**
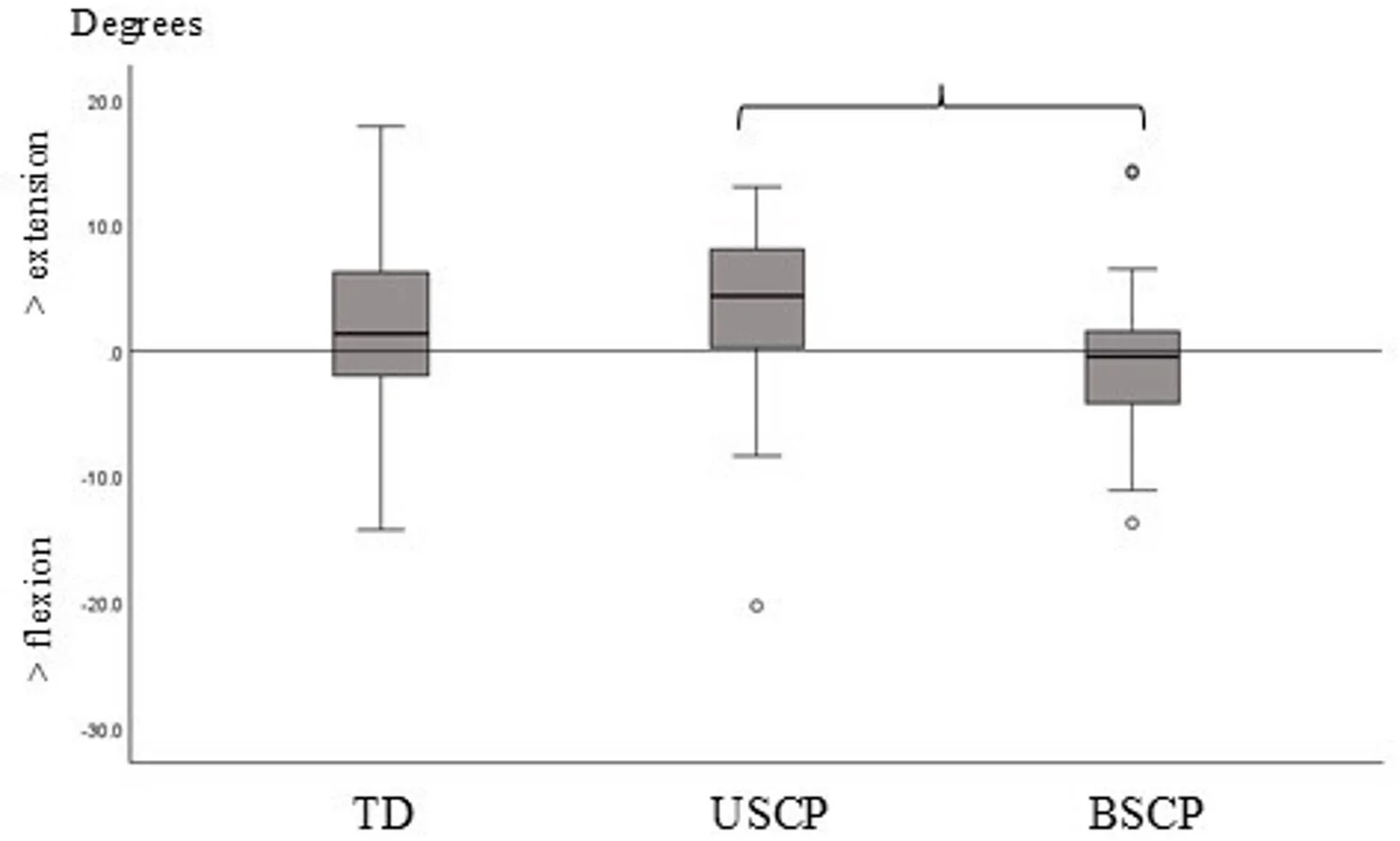
Illustration of joint position sense errors, the relative difference between the criterion angle (CA) and the reproduced angle (RA) indicating the direction of the error (positive value= a more extended knee, negative value= a more flexed knee) presented for the typically developing children (TD) and for the children with unilateral spastic cerebral palsy (USCP) and bilateral spastic CP (BSCP) as median [min, max] degrees. Differences between the groups are marked with brackets

The capacity to reproduce the knee angle, i.e., joint position sense, did not differ between the group with CP and the TD group. With respect to spastic subtype, absolute JPS errors, median [min, max] degrees, were greater for the children with USCP; 6.6 [0.2, 20.3] compared to the children with BSCP; 3.6 [0.2, 14.4], (*p* = 0.037). The group with USCP had a more extended knee than the group with BSCP (*p* = 0.012) (Fig. 3).

Age was negatively associated with absolute JPS errors in the group with CP (r_s_=-0.366, *p* = 0.006), indicating that older participants tended to demonstrate more accurate JPS (lower errors). There was no association between age and JPS errors for the TD group (r_s_=-0.244, *p* = 0.119).

### Maintaining the knee angle position

#### The passively induced criterion angle trial

The duration from the passively induced CA to strap release, median [min, max] seconds, was longer for the entire group of children with CP; 4.0 [1.1, 8.3] compared to TD peers; 2.6 [0.5, 5.7] (*p* < 0.001), and for the children at GMFCS III-IV; 5.5 [1.1, 7.2] compared to GMFCS I; 3.3 [1.4, 6.7] (*p* = 0.036). Duration did not differ between the spastic subtypes (USCP vs. BSCP).

**Fig. 4:**
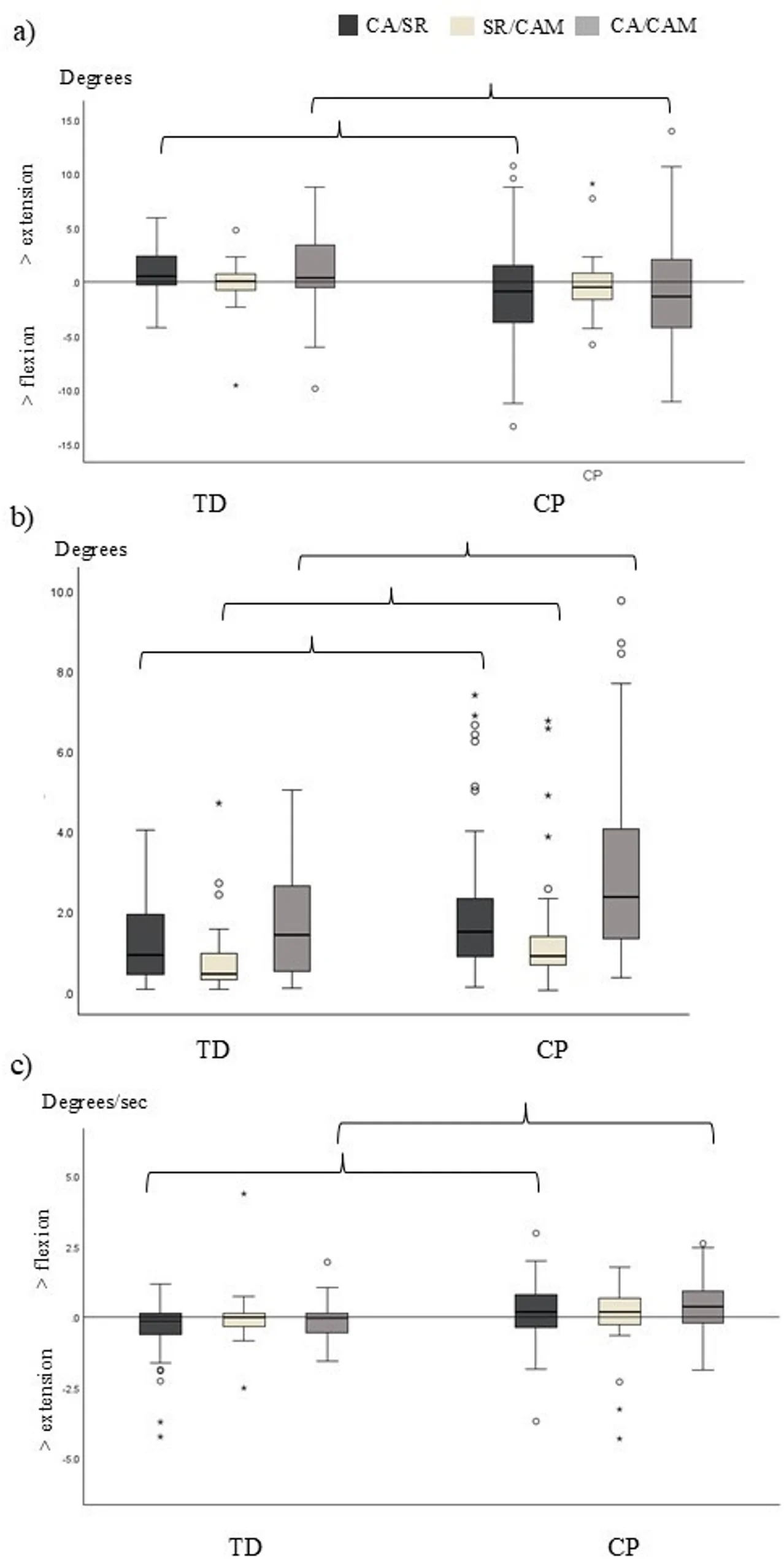
Illustration of the capacity to maintain the knee angle in the passively induced criterion angle (CA) trial: CA/strap release (SR), SR/CA maintained 2.5 sec (CAM), and CA/CAM for the children typically developing (TD) and those with cerebral palsy (CP) presented as: (**a**) differences in the direction of the knee angle in degrees (positive value= a more extended knee, negative value= a more flexed knee). **b **fluctuations in degrees (0= no fluctuations). **c** drift over time in degrees/seconds (positive value= a more flexed knee, negative value= a more extended knee). 0 degrees = stable position. Differences between groups are marked with brackets

When maintaining the knee angle during the passively induced CA trial the differences in knee angle were greater for the group with CP than the TD group at: CA vs. SR (*p* = 0.002), SR vs. CAM (*p* = 0.031) and CA vs. CAM (*p* = 0.010) (Supplementary Table 1). The knee was more flexed in the CP group compared to the TD group at: CA vs. SR (*p* < 0.001) and at CA vs. CAM (*p* = 0.006) (Fig. 4a).

Fluctuation of the knee position over time was greater for the group with CP than the TD group at the intervals: CA-SR (*p* = 0.031), SR-CAM (*p* = 0.005), and CA-CAM (*p* = 0.006) (Fig. 4b). Regarding drift of the position over time, the leg drifted towards increased knee flexion in CA-SR (*p* < 0.001) and CA-CAM (*p* = 0.006) for the group with CP compared to the TD group (Fig. 4c). Between subgroups (USCP vs. BSCP or GMFCS groups; GMFCS I, GMFCS III, GMFCS III-IV) the results did not differ significantly on any measure (Supplementary Tables 1, and Supplementary Table 2).

#### The actively induced reproduced angle trial

When maintaining the knee position during the actively induced RA trial (RA-RAM) there was a drift towards knee extension in the TD group, but not in the group with CP (*p* = 0.025). Between subgroups (USCP vs. BSCP or GMFCS groups; GMFCS I, GMFCS III, GMFCS III-IV) the results did not differ significantly on any measure (Supplementary Tables 1 and Supplementary Table 2).

#### Within-group results comparing the passively induced angle trial with the actively induced reproduced angle trial

##### CA/CAM compared with RA/RAM

**Fig. 5:**
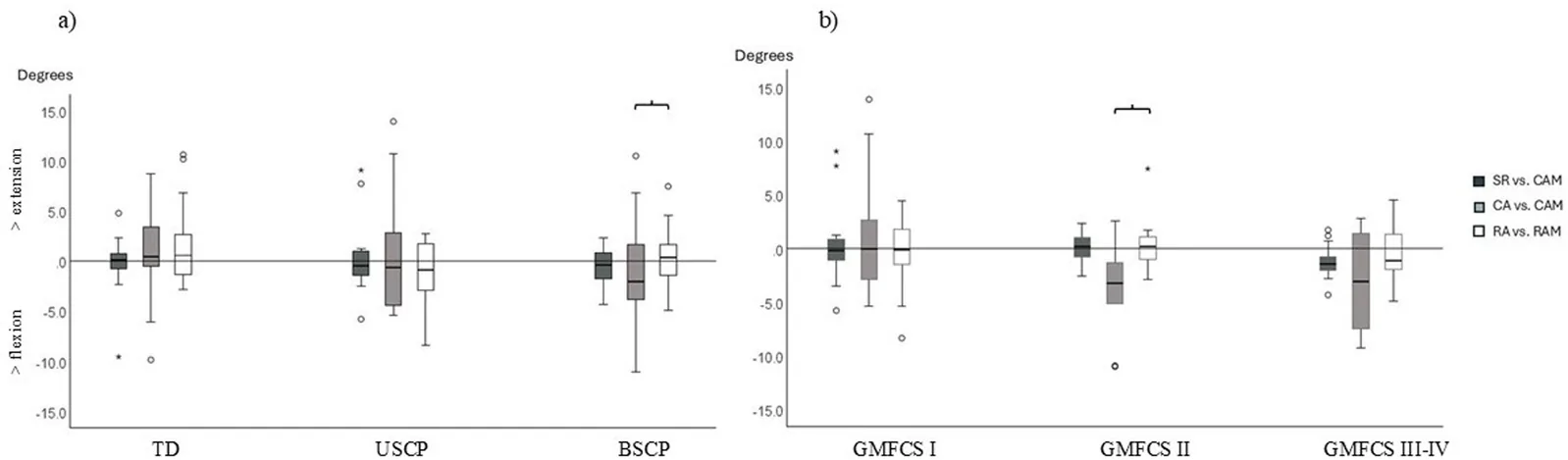
Illustration of within-group differences in knee angles (degrees) when maintaining the leg in a joint position sense test. The passively induced criterion angle (CA) trial; Strap release (SR) vs. CA maintained 2,5 seconds (CAM), and CA vs. CAM were compared with the actively reproduced angle (RA) trial: RA vs. RA maintained 2.5 seconds (RAM) for the groups of children: (**a**) typically developing (TD), unilateral spastic cerebral palsy (USCP), bilateral spastic cerebral palsy (BSCP). **b **with CP at Gross Motor Functioning Classification System (GMFCS) I, II, and III-IV. 0 degrees = stable position. Negative value =a more flexed knee, a positive value = a more extended knee. Brackets indicate significant differences between the passively induced CA-trial and the actively induced RA-trial within the group

When comparing the passively induced trial; CA vs. CAM with the actively induced trial RA vs. RAM the knee angle differed more at CA vs. CAM within the groups of children with BSCP (*p* = 0.001), GMFCS II (*p* = 0.013), and GMFCS III-IV (*p* = 0.033), and knee was more flexed in the BSCP group (*p* = 0.015) (Fig. 3a) and the GMFCS II group (*p* = 0.028) (Fig. 5a and b).

Fluctuation of the knee position over time was greater in the interval CA-CAM compared to RA-RAM in the group with USCP (*p* = 0.012), BSCP (*p* = 0.008), GMFCS I (*p* = 0.012), and GMFCS II (*p* = 0.017). A drift towards increased knee flexion over time was found in the BSCP group in CA-CAM compared to RA-RAM (*p* = 0.040).

##### SR/CAM compared with RA/RAM

When comparing the passively induced trial; SR vs. CAM with the actively induced trial RA vs. RAM, the knee angle differed more at RA vs. RAM in the TD group (*p* < 0.001), and in the group at GMFCS I (*p* = 0.046). However, there was no difference with regard to direction (Fig. 5a, and b).

Fluctuation of the knee position over time was greater in the interval SR-CAM compared to RA-RAM in the TD group (*p* < 0.001), and in RA-RAM compared to SR-CAM in the group at GMFCS I (*p* = 0.015). Drift in knee position over time did not differ within any of the groups.

## Discussion

This study investigated proprioception in the knee from new perspectives, including both the capacity to reproduce and maintain a knee position in children with spastic CP. Consistent with one of our hypotheses, the capacity to reproduce a knee angle with accuracy, i.e. joint position sense, was similar for the group of children with CP and the TD children. However, in the subgroup with USCP, the position was overestimated, with a more extended knee. Consistent with our second hypothesis, maintaining a passively induced knee position was challenging for the group with CP, especially for the subgroup with BSCP, and for those with walking limitations, in whom the knee was more flexed. The difficulties mainly occurred in the interval including the transition from examiner-supported to self-maintained limb positioning i.e., involving the strap release. Moreover, in the group with BSCP and in those at GMFCS II the knee angle drifted towards greater flexion over time in the passively induced CA trial compared to in the actively induced RA trial.

The results revealed a divergence in proprioceptive function related to the subtype of spastic CP. The children with USCP had difficulties with reproducing a knee angle, measured as JPS errors. In contrast, the children with BSCP had difficulty maintaining knee position when the leg was passively positioned. The difficulties in maintaining the passively induced knee angle, and adapt to the release of the strap, could indicate difficulties with processing of sensory information from the proprioceptive system important for motor planning. Further investigations in a weight-bearing position are warranted to explore whether the observed drift towards knee flexion in sitting could contribute to the flexed knee position during standing and walking previously described in children with BSCP at GMFCS level II-III [6, 32]. Aberrant somatosensory cortical activity in youths with BSCP during static ankle plantarflexor force generation in sitting has been related to walking velocity and step length [33].

The prolonged duration from the criterion angle to strap release for the children with CP raised questions among the examiners during the testing. Muscle spindles provide rapid sensory feedback about stretch and movement, and timely muscle activation is required for this information to be effectively used. Delays in activation may therefore reduce the efficiency of responses to sensory input [34]. The extended duration could reflect deficits in motor control, specifically difficulties in adapting motor output to environmental task demands [1]. Delayed motor execution has been associated with altered sensorimotor processing in youths with BSCP and may reflect broader cognitive-perceptual challenges [35]. Reduced attention span, which is commonly described in individuals with CP [2] and the interaction with the examiner could also contribute to the prolonged duration.

Building on our previous study using the JPS test on children with BSCP [27], children with USCP were included in this study, and a more comprehensive analysis of the capacity to maintain the static position of the leg was conducted. The decision to focus solely on the knee flexion angle of 45 degrees and to perform data analysis on one leg for each child was based on similar results in JPS errors for both legs for the same cohort of children with BSCP and TD for three tested angles (20, 45 and 70 degrees) in the previous study [27]. Additionally, the 45-degree knee angle was selected because it had the most data in the previous study on the BSCP group, whereas more data were missing at the other angles due to marker occlusion and inability to maintain the leg´s position. In the group with USCP, analyses were confined to the most affected leg to evaluate the influence of spastic subtype on the outcomes rather than potential bilateral sensorimotor alterations.

Methodological differences across studies exploring various aspects of proprioception in heterogeneous populations make it difficult to compare results. In the present study, and the study by McLaughlin et al. [25], comparable JPS errors were found between children with CP and TD children. In contrast, Marsico et al. [24] reported on greater JPS errors in children with upper motor neuron lesions, including children with CP, than in the TD group. In the JPS test used by Marsico and colleagues, the reproduced angle was not self-assumed by the child; instead, the assessor moved the leg, and the child indicated when they perceived their leg had reached the target. Moreover, inertial motion capture was used instead of 3DMA. The difference in method and sample impedes comparisons of the results.

The JPS test used in this study was performed in a controlled environment [26, 27], and the test can be described as a low-level task primarily assessing proprioceptive capacity using an egocentric spatial frame of reference [36]. Even though it is a more demanding test than simple movement detection testing. In clinical practice, it is recommended to combine low-level tests with high-level functional proprioceptive tests. High-level proprioceptive tests are performed with an allocentric frame of reference, in which positions and movements are represented relative to external landmarks or the environment rather than the body itself [36]. An example would be an obstacle clearance test: stepping over a hurdle-shaped obstacle, with downward vision occluded, aiming for minimal clearance [37]. The heterogeneous central deficits in individuals with CP may influence proprioceptive performance, as proprioception relies on both peripheral afferent inputs and central signals derived from efferent motor commands, thereby enabling a central prediction of the movement [36, 38]. Consequently, a high-level proprioceptive test that requires more complex neural processing, recruiting additional brain areas [36], might give further information. Using a high-level proprioceptive test for research purposes can be challenging since multiple factors must be controlled for. However, complexity offers opportunities for methodological advances. In adult athletes, proprioception has been assessed with obstacle clearance tests [37]. For children with CP who, per se, have limitations in motor functioning, this test is complex and could constitute a fall risk. There is a need for a functional, child-friendly test to assess proprioception in the lower extremities of children with CP.

### Study strengths and limitations

A methodological strength of this study was that the second of two trials was used for data-analysis allowing all participants to use the learning effect during testing [39]. Moreover, using automatically calculated events, rather than setting events manually, promotes objectivity, consistency, and reproducibility of results.

The number of missing data (9%, 5 of 37) on knee angle at CAM in the passively induced criterion angle trial in the BSCP group reflects both limited ability to maintain knee position among the children and marker occlusion, which may have introduced selection bias. A possible limitation of the JPS test used was that an examiner passively elevated children’s legs to the CA. Manual movements by an examiner cannot ensure uniform velocity and termination at the precise, predetermined joint angle. In future studies, robot-aided movement assessments could enhance the sensitivity of proprioceptive measurements [40]. Another limitation of the JPS test used was that visual information from the legs was occluded throughout. Providing visual information at one trial and occluding vision at another could clarify whether JPS errors emerge only in the absence of visual input or are present even when visual input is available. Modifying the protocol could allow direct comparison between the two conditions, possibly providing more information. Adding surface electromyography to the analysis could also shed light on possible co-activation of muscles described in children with CP [41] which could interfere with maintaining the leg in a stable position.

Generalizations of the results should be made with caution. The participants cannot be considered representative of the entire population with spastic CP, as they were relatively highly functioning: most were at GMFCS levels I-II, although children at GMFCS levels III-IV were represented, and all were able to understand the instructions. Moreover, the method for evaluating the children´s capacity to maintain the leg position is innovative and would benefit from validation in a larger cohort.

## Conclusion

The results reveal a divergence in proprioceptive function: while children with USCP had more difficulties than children with BSCP in reproducing a knee angle (i.e., greater JPS errors), the children with BSCP demonstrated greater difficulty in maintaining the knee position, most apparent during passive positioning of the lower limb. The findings provide insights into the sensorimotor challenges of spastic CP. Increased knowledge of lower-limb proprioception in children with CP could inform the development of innovative interventions, tailored to subtype and motor functioning, to optimize postural control and motor development.

## Supplementary Information

Supplementary Material 1.

Supplementary Materials 2. Table 1 and 2

## Data Availability

The datasets used and analysed during the current study are available from the corresponding author on reasonable request.

## Abbreviations

BSCP: Bilateral spastic cerebral palsy
CA: Criterion angle
CAM: Criterion angle maintained
CP: Cerebral palsy
GMFCS: Gross Motor Function Classification System
JPS: Joint position sense
RA: Reproduced angle
RAM: Reproduced angle maintained
RMSE: Root mean square error
SR: Strap release
TD: Typical development
USCP: Unilateral spastic cerebral palsy
3DMA: Three-dimensional motion analysis
