# Supplementary material for: Knee proprioception in children with spastic cerebral palsy: an observational study
Source: BMC Pediatr. 2026 Sep 15;26:871. doi: 10.1186/s12887-026-07652-2 (PMC13579802; doi:10.1186/s12887-026-07652-2)
Supplement: Supplementary file 2 [file 12887_2026_7652_MOESM2_ESM.docx]

| Supplementary Materials  Table 1. Differences between the groups of children with typical development (TD) vs. spastic cerebral palsy (CP), and between the spastic subtypes, unilateral spastic CP (USCP) vs. bilateral spastic CP (BSCP). Joint-position sense (JPS): the absolute and relative difference between Criterion Angle (CA) and Reproduced Angle (RA). Difference in knee angle and direction (negative value = a more flexed knee, positive value= a more extended knee), fluctuation and drift of the position (negative value=drift towards extension, positive value= drift towards more flexion) between the events; CA/SR, SR/CA maintained 2.5 seconds (CAM), CA/CAM, and RA/RA maintained 2.5 seconds (RAM) presented as median [minimum, maximum] values. Zero degrees indicates a maintained position. The “*n*” indicates the number of valid measures for each calculation. Bold values indicate significant differences between the groups (*p*<0.05). | | | | | | | | | | | |
| --- | --- | --- | --- | --- | --- | --- | --- | --- | --- | --- | --- |
|  | TD | | CP | | Difference  TD vs. CP | USCP | | BSCP | | Difference  USCP vs BSCP | |
|  | *n* | median  [min, max] | *n* | median  [min, max] | *p-value* | *n* | median  [min, max] | *n* | median  [min, max] | | *p-value* |
| **JPS error** |  |  |  |  |  |  |  |  |  | |  |
| JPS absolute error, degrees | 42 | 4.2 [0.5, 17.9] | 56 | 4.3 [0.2, 20.3] | 0.909 | 19 | 6.6 [0.2, 20.3] | 37 | 3.6 [0.2, 14.4] | | **0.037** |
| JPS relative error, degrees | 42 | 1.3 [-14.2, 17.9] | 56 | 0.6 [-20.3, 14.4] | 0.245 | 19 | 4.4 [-20.3, 13.1] | 37 | -0.5 [-13.7, 14.4] | | **0.012** |
| **CA/SR** |  |  |  |  |  |  |  |  |  | |  |
| Diff. in knee angle, degrees | 42 | 1.1 [0.0, 8.6] | 57 | 3.2 [0.1, 13.4] | **0.002** | 20 | 4.0 [0.6, 9.6] | 37 | 2.3 [0.1, 13.4] | | 0.160 |
| Diff. in knee angle, direction, degrees | 42 | 0.8 [-4.2 ,8.6] | 57 | -1.2 [-13.4, 10.7] | **<0.001** | 20 | -1.9 [-5.4, 9.6,] | 37 | -1.2 [-13.4, 10.7] | | 0.525 |
| Fluctuation of knee angle position, degrees | 42 | 1.0 [0.1, 7.5] | 57 | 1.5 [0.1, 7.4] | **0.031** | 20 | 1.6 [0.3, 7.4] | 37 | 1.4 [0.1, 6.9] | | 0.442 |
| Drift of knee angle position, degrees/s | 42 | -0.2 [-8.5, 1.2] | 57 | 0.3 [-3.7, 3.0] | **<0.001** | 20 | 0.3 [-1.9, 1.1] | 37 | 0.3 [-3.7, 3.0] | | 0.628 |
| **SR/CAM** |  |  |  |  |  |  |  |  |  | |  |
| Diff. in knee angle, degrees | 40 | 0.8 [0.0, 9.6] | 51 | 1.2 [0.0, 9.1] | **0.031** | 19 | 1.1 [0.0, 9.1] | 32 | 1.2 [0.0, 4.3] | | 0.755 |
| Diff. in knee angle, direction, degrees | 40 | 0.1 [- 9.6, 4.8] | 51 | -0.5 [-5.8, 9.1] | 0.117 | 19 | -0.7 [-5.8, 9.1] | 32 | -0.4 [- 4.3, 2.3] | | 0.815 |
| Fluctuation of knee angle position, degrees | 40 | 0.5 [0.1, 4.7] | 51 | 0.9 [0.0, 6.8] | **0.005** | 19 | 1.0 [0.2, 6.8] | 32 | 0.9 [0.0, 4.9] | | 0.381 |
| Drift of knee angle position, degrees/s | 40 | 0.0 [-2.5, 4.4] | 51 | 0.2 [-4.3, 1.8] | 0.073 | 19 | 0.2 [-4.3, 1.4] | 32 | 0.1 [-3.3, 1.8] | | 0.770 |
| **CA/CAM** |  |  |  |  |  |  |  |  |  | |  |
| Diff. in knee angle, degrees | 40 | 1.7 [0.0, 9.9] | 51 | 3.1 [0.0, 13.9] | **0.010** | 19 | 3.6 [0.0, 14.0] | 32 | 3.0 [0.2, 11.0] | | 0.654 |
| Diff. in knee angle, direction, degrees | 40 | 0.4 [-9.9, 8.7] | 51 | -1.3 [-11.0, 13.9] | **0.006** | 19 | -0.9 [-6.8, 14.0] | 32 | -2.0 [-11.1, 10.5] | | 0.340 |
| Fluctuation of knee angle position, degrees | 40 | 1.4 [0.1, 5.0] | 51 | 2.4 [0.4, 9.7] | **0.006** | 19 | 2.8 [0.6, 8.4] | 32 | 2.2 [0.4, 9.7] | | 0.250 |
| Drift of knee angle position, degrees/s | 40 | -0.1 [-1.6, 1.9] | 51 | 0.4 [-1.9, 2.6] | **0.006** | 19 | 0.3 [-1.7, 1.3] | 32 | 0.4 [-1.9, 2.6] | | 0.320 |
| **RA/RAM** |  |  |  |  |  |  |  |  |  | |  |
| Diff. in knee angle, degrees | 42 | 1.7 [0.0, 10.6] | 56 | 1.7 [0.1, 8.4] | 0.730 | 19 | 1.8 [0.7, 8.4] | 37 | 1.5 [0.1, 7.5] | | 0.139 |
| Diff. in knee angle, direction, degrees | 42 | 0.2 [-2.9. 10.6] | 56 | -0.2 [-8.4, 7.5] | 0.124 | 19 | -1.0 [-8.4. 2.7] | 37 | 0.3 [-4.9, 7.5] | | 0.257 |
| Fluctuations of knee angle position, degrees | 42 | 1.3 [0.3, 6.7] | 56 | 1.3 [0.2, 21.7] | 0.983 | 19 | 1.6 [0.8, 5.2] | 37 | 1.2 [0.2, 21.7] | | 0.144 |
| Drift of knee angle position, degrees/s | 42 | -0.3[-4.8, 2.0] | 56 | 0.1 [-2.6, 7.6] | **0.025** | 19 | 0.4 [-1.3, 3.2] | 37 | 0.0 [-2.6, 7.6] | | 0.279 |

| Table 2. Differences between the groups of children functioning at Gross Motor Functioning Classification System (GMFCS) I, GMFCS II, and GMFCS III-IV. Joint position sense (JPS) as the relative and absolute difference (error) between Criterion Angle (CA) and Reproduced Angle (RA). Difference in knee angle and direction (negative value = a more flexed knee, positive value= a more extended knee), fluctuation and drift of the position (negative value=drift towards extension, positive value= drift towards more flexion) between the events; CA/SR, SR/CA maintained 2.5 seconds (CAM), CA/CAM, and RA/RA maintained 2.5 seconds (RAM) presented as median [minimum, maximum] values. The “*n*” indicates the number of valid measures for each calculation. Bold values indicate significant differences between the groups (*p*<0.05). In post hoc tests, the Bonferroni correction hasSupplementary adjusted significant values for multiple comparisons, as indicated with **^¤^**. N/A= not applicable. | | | | | | | | | | |
| --- | --- | --- | --- | --- | --- | --- | --- | --- | --- | --- |
|  | GMFCS I GMFCS II GMFCS III-IV | | | | | | Difference  I, II, III-V | I vs  II | I vs  III-IV | II vs  III-IV |
|  | *n* | median  [min, max] | *n* | median  [min, max] | *n* | median  [min, max] | *p-value* | *p-value* | *p-value* | *p-value* |
| **JPS error** |  |  |  |  |  |  |  |  |  |  |
| JPS absolute error, degrees | 30 | 5.3 [0.2, 20.3] | 11 | 3.6 [0.3, 11.0] | 15 | 2.9 [0.4, 14.4] | 0.749 | N/A | N/A | N/A |
| JPS relative error, degrees | 30 | 1.1 [-20.3, 14.1,] | 11 | 0.3 [-11.1, 0.5] | 15 | -1.0 [-13.7, 4.4] | 0.350 | N/A | N/A | N/A |
| **CA/SR** |  |  |  |  |  |  |  |  |  |  |
| Diff. in knee angle, degrees | 31 | 3.7 [0.2, 10.7] | 11 | 2.7 [0.2, 13.4] | 15 | 3.2 [0.1, 8.7] | 0.747 | N/A | N/A | N/A |
| Diff. in knee angle, direction, degrees | 31 | -0.7 [-10.7, 10.7] | 11 | -2.7 [-13.4, 1.0] | 15 | -1.6 [-8.7, 4.2] | 0.058 | N/A | N/A | N/A |
| Fluctuations of knee angle position, degrees | 31 | 1.5 [0.1, 7.4] | 11 | 1.2 [0.1, 6.4] | 15 | 1.4 [0.3, 5.0] | 0.825 | N/A | N/A | N/A |
| Drift of knee angle position, degrees/s | 31 | 0.1 [-3.7, 1.6] | 11 | 0.4 [-0.1, 3.0] | 15 | 0.3 [-0.6, 1.5] | 0.239 | N/A | N/A | N/A |
| **SR/CAM** |  |  |  |  |  |  |  |  |  |  |
| Diff. in knee angle, degrees | 28 | 1.0 [0.0, 9.1] | 10 | 0.9 [0.1, 2.6] | 13 | 1.7 [0.7, 4.3] | 0.128 | N/A | N/A | N/A |
| Diff. in knee angle, direction, degrees | 28 | -0.2 [-5.8, 9.1 ] | 10 | 0.2 [-2.6, 2.3] | 13 | -1.5 [-4.3,1.7] | 0.129 | N/A | N/A | N/A |
| Fluctuation of knee angle position, degrees | 28 | 0.9 [0.1, 6.8] | 10 | 1.1 [0.0, 3.9] | 13 | 1.2 [0.5, 4.9] | 0.190 | N/A | N/A | N/A |
| Drift of knee angle position, degrees/s | 28 | 0.1 [-4.3, 1.7] | 10 | 0.0 [-3.3, 0.9] | 13 | 0.5 [-0.6, 1.8] | 0.342 | N/A | N/A | N/A |
| **CA/CAM** |  |  |  |  |  |  |  |  |  |  |
| Diff. in knee angle, degrees | 28 | 2.9 [0.0, 13.9] | 10 | 3.3 [0.3, 11.0] | 13 | 3.1 [1.3, 9.3] | 0.803 | N/A | N/A | N/A |
| Diff. in knee angle, direction degrees | 28 | -0.3 [-6.8, 13.9] | 10 | -3.3 [-11.1, 2.6] | 13 | -3.1 [-9.3, 2.8] | **0.033** | 0.082**^¤^** | 0.142**^¤^** | 1.000**^¤^** |
| Fluctuation of knee angle position, degrees | 28 | 2.6 [0.4, 8.7] | 10 | 2.3 [0.4, 9.7] | 13 | 2.2 [0.7, 6.6] | 0.956 | N/A | N/A | N/A |
| Drift of knee angle position, degrees/s | 28 | 0.0 [-1.9, 1.3] | 10 | 0.6 [-0.1, 2.6] | 13 | 0.5 [-0.6, 2.5] | **0.045** | 0.089**^¤^** | 0.222**^¤^** | 1.000**^¤^** |
| **RA/RAM** |  |  |  |  |  |  |  |  |  |  |
| Diff. in knee angle, degrees | 30 | 1.8 [0.1, 8.4] | 11 | 1.0 [0.2, 7.5] | 15 | 1.9 [0.3, 4.9] | 0.252 | N/A | N/A | N/A |
| Diff. in knee angle, direction, degrees | 30 | -0.7 [-8.4, 4.4,] | 11 | 0.5 [-2.9. 7.5] | 15 | 0.3 [-4.9, 4.6] | 0.851 | N/A | N/A | N/A |
| Fluctuation of knee angle position, degrees | 30 | 1.4 [0.2, 5.2] | 11 | 1.1 [0.4, 6.6] | 15 | 1.4 [0.4, 21.7] | 0.568 | N/A | N/A | N/A |
| Drift of knee angle position, degrees/s | 30 | 0.1 [-2.1, 3.2] | 11 | 0.1 [-2.6, 1.5] | 15 | 0.2 [-1.8, 7.6] | 0.804 | N/A | N/A | N/A |
